# Postmortem Analysis of Opioids and Metabolites in Skeletal Tissue

**DOI:** 10.1093/jat/bkab095

**Published:** 2021-09-05

**Authors:** Michiel Vandenbosch, Stane Pajk, Wouter Van Den Bogaert, Joke Wuestenbergs, Wim Van de Voorde, Eva Cuypers

**Affiliations:** KU Leuven, Toxicology and Pharmacology, Department of pharmaceutical sciences, Campus Gasthuisberg, Onderwijs en Navorsing 2, Herestraat 49, Leuven 3000, Belgium; Maastricht University, M4I Institute, Division of Imaging Mass Spectrometry, Universiteitssingel 50, 6229 ER Maastricht, The Netherlands; KU Leuven, Toxicology and Pharmacology, Department of pharmaceutical sciences, Campus Gasthuisberg, Onderwijs en Navorsing 2, Herestraat 49, Leuven 3000, Belgium; University of Ljubljana, Faculty of Pharmacy, Aškerčeva 7, SI-1000 Ljubljana, Slovenia; KU Leuven, Imaging and Pathology Department, Division Forensic Biomedical Sciences, Campus Sint-Rafaël, Kapucijnenvoer 33, 3000 Leuven, Belgium; KU Leuven, Imaging and Pathology Department, Division Forensic Biomedical Sciences, Campus Sint-Rafaël, Kapucijnenvoer 33, 3000 Leuven, Belgium; KU Leuven, Imaging and Pathology Department, Division Forensic Biomedical Sciences, Campus Sint-Rafaël, Kapucijnenvoer 33, 3000 Leuven, Belgium; KU Leuven, Toxicology and Pharmacology, Department of pharmaceutical sciences, Campus Gasthuisberg, Onderwijs en Navorsing 2, Herestraat 49, Leuven 3000, Belgium; Maastricht University, M4I Institute, Division of Imaging Mass Spectrometry, Universiteitssingel 50, 6229 ER Maastricht, The Netherlands

## Abstract

Every year, thousands of suspicious deaths are accounted for by an overdose of opioids. Occasionally all traditional matrices are unavailable due to decomposition. Skeletal tissue may pose a valid alternative. However, reference data on postmortem concentrations in bone tissue and bone marrow (BM) is sparse. Therefore, a liquid chromatography--tandem mass spectrometry method was developed and fully validated for the analysis of four opioids and two metabolites (tramadol, O-desmethyltramadol, morphine, fentanyl, norfentanyl, codeine) in bone tissue and BM. Sample preparation was performed using solid phase extraction (BM), methanolic extraction (bone) and a protein precipitation (whole blood). All validation parameters were successfully fulfilled. This method was applied to analyze 22 forensic cases involving opioids. All six opioids were proven to be detectable and quantifiable in all specimens sampled. When tramadol blood concentrations were correlated with bone concentrations, a linear trend could be detected. The same was seen between tramadol blood and BM concentration. A similar linear trend was seen when correlating codeine blood concentration with bone and BM concentration. Although some variability was detected, the same linear trend was seen for morphine. For fentanyl and norfentanyl, the sample size was too small to draw conclusions, regarding correlation. As far as the authors know this is the first-time fentanyl and norfentanyl are quantified in skeletal tissue. In conclusion, due to the absence of reference data for drugs in skeletal tissue, these findings are a step forward toward a more thorough understanding of drug concentration found in postmortem skeletal tissue.

## Introduction

Opioids are the most (ab)used drugs in the world today (1). They exhibit selective binding to receptors at several sites in the central nervous system. This makes them extremely useful in the medical world where they are used as anesthetics and painkillers (2). Sadly, most of the opioids are also widely abused. The analgesic effect can produce euphoria in some instances. This feeling of euphoria makes the opioid drugs attractive for recreational usage (1). Due to the inhibiting effects of these drugs on the central nervous system, risks are involved when using these drugs. These risks include a chance of hypotension, hypothermia, pulmonary effects, gastrointestinal effects, seizures and even eventually coma (2). As a result, in Europe 85% of the deaths due to a drug overdose can be accounted for by one or more opioids (3).

In forensic toxicology, usually body fluids are used for analysis (4). For blood as well as for urine, a lot of reference material is available to determine correlation between drug concentration and effect. However, in some cases when extended time has elapsed before a body is discovered, blood and urine are not available anymore (5). Since skeletal tissue withstands degradation, it may pose a valid alternative when other specimens are degraded. Starting from the seventies, multiple case reports can be found describing the detection of drugs in skeletal tissue (6, 7). Most of them use bone marrow (BM), since marrow is highly vascularized and well protected by bone. Many studies show BM to act as an excellent drugs depot (8, 9). A discrepancy exists between studies regarding correlation between drug blood concentration and BM concentration. Several studies using animal models show a good correlation between blood concentration and BM concentration (10, 11). Nevertheless, almost an equal amounts of studies show poor correlation (8, 12).

When looking at publications regarding bone tissue, a similar trend is seen. Starting from the seventies, a paucity of studies has been published regarding detection of drugs in human bone tissue (13, 14). The last decade, several studies have been undertaken using animal models to study drug distribution in bone tissue (15–17). Multiple drugs have been detected in bone tissue. In addition, some parts of the bone prove to be more suitable for analysis than others. Parts containing more vascularization like trabecular bone tend to give higher concentrations (18). The same is true when comparing whole bones. Bones with a higher vascularization rate resulted in higher concentrations (19). No correlation could be found between blood concentration and bone tissue in animal studies but there are some indications for a correlation between administered dose and bone drug concentration (19).

Using animal models, it is possible to broadly study any correlation between drug concentrations in peri/postmortem skeletal tissue with considerably greater statistical power. Nevertheless, obtained data using animals cannot be extrapolated to casework involving humans (20). Different authors have used a variety of methodologies for drug extraction and detection but studies performed using human bones are still scarce. To-date there is no standardized protocol for sample preparation and analysis. Despite the considerable renewal of interest in the topic over the last 20 years, the drug concentration found in skeletal tissue of postmortem human samples remains poorly understood. One of the possible reasons for the lack of research on bone (marrow) is the difficulty in obtaining bones from cases. In a previous project, a linear trend could be found in human autopsy samples between blood and bone concentration for methadone (21). When methadone concentrations in blood and BM were compared, an exponential trend could be seen. This study aims to expand this study to other opioids in order to increase the understanding of drug concentration in skeletal tissue. In term, this will increase the utility of this matrix in toxicological examinations by collecting reference data.

## Materials and Methods

### Chemicals and reagent

Analytical reference standards of tramadol (1 mg/mL), cis-tramadol-13 C-d_3_ (100 µg/mL), O-desmethyltramadol (1 mg/mL), morphine-d_3_ (100 µg/mL), morphine (1 mg/mL), norfentanyl (1 mg/mL), norfentanyl-d_5_ (100 µg/mL), fentanyl (1 mg/mL) fentanyl-d_5_ (1 mg/mL), codeine (1 mg/mL) and codeine-d_3_ (100 µg/mL) were purchased from LGC standards (Teddington, UK). For making calibrators, reference standards were mixed to different concentrations in methanol. Separate methanolic standard stock solutions of deuterated analogues and the 13C-labeled analogue were prepared. All standard solutions were stored at −20°C.

All solvents, chemicals and reference standards were at least of analytical or HPLC grade. Acetonitrile and methanol were obtained from Biosolve (Valkenswaard, The Netherlands). Dichloromethane, acetic acid, 2-propanol, monopotassium phosphate and ammonium hydroxide that were used in the sample preparation were purchased from Merck (Darmstadt, Germany).

Formic acid and ammonium formate were purchased from Sigma-Aldrich (Bornem, Belgium). Deionized water was prepared using a Milli-Q Water Purification System (Millipore, Brussels, Belgium). The aqueous buffer was prepared by adding 10 mM ammonium bicarbonate and setting the pH at 9.0 with ammonium hydroxide.

Bond Elut Plexa PCX cartridges (60 mg, 3 mL) were purchased from Varian (Sint-Katelijne-Waver, Belgium). All solid phase extractions (SPEs) were carried out on a Vac Elut SPS 24 (Varian, Sint-Katelijne-Waver, Belgium).

### Sample collection

During the period from April 2018 to March 2020, samples were obtained during autopsies of legal cases at UZ Leuven (Belgium). The clavicle bone was chosen as specimen of choice due to the high accessibility during autopsy. For each case, the case background and the medical history was reported as provided by the legal system. A brief summary including postmortem intervals can be found in Supplementary Table S1. Cases were selected after a positive screening result for tramadol, morphine, fentanyl, codeine, cocaine or any of their metabolites in blood using the method as described by Vandenbosch et al. (21). Twenty-two cases were selected. Six females and 16 males were analyzed. The age of the deceased ranged from 21 to 83 years old with a median of 40 years old. Samples were collected and treated as described in Vandenbosch et al. (21). Blood was sampled from the vena saphena magna. Approval for this study was received from the Medical Ethics Committee of the faculty of Medicine of the University Hospital of Leuven, Belgium.

### Specimen preparation

Blood samples were extracted using a simple protein precipitation procedure as reported by our group (19, 22). One-hundred microliters of this blood was spiked with 10 μL of codeine-d_3_ (1 μg/mL), 10 μL of morphine-d_3_ (1 μg/mL), 10 μL of cis-tramadol-C13-d_3_ (1 μg/mL), 10 μL of fentanyl-d_5_ (1 μg/mL) and 10 μL of norfentanyl-d_5_ (1 μg/mL) followed by a protein precipitation. A ring of 1 cm width was serrated 1 cm from the center of the proximal clavicle head. More information can be found in the supplementary SOP. The bone samples were extracted using a methanolic extraction as recently reported by our group (19, 22). At the start of the extraction, single full bones were spiked with internal standards (IS) by addition 50 ng of codeine-d_3_, 50 ng of morphine-d_3_, 50 ng of cis-tramadol-C13-d_3_, 50 ng of fentanyl-d_5_ and 50 ng of norfentanyl-d_5_ in the extraction solvent. BM was extracted using SPE as described by our group (21). The 100 mg of BM was mixed with 10 μL of codeine-d_3_ (10 μg/mL), 10 μL of morphine-d_3_ (10 μg/mL), 10 μL of cis-tramadol-C13-d_3_ (10 μg/mL), 10 μL of fentanyl-d_5_ (10 μg/mL) and 10 μL of norfentanyl-d_5_ (10 μg/mL).

### LC--MS-MS method

Separation of the compounds was performed on a Shimadzu Prominence Ultra-Fast Liquid Chromatograph XR System (Shimadzu Benelux, Jette, Belgium) in combination with an Acquity UPLC^®^ BEH C18 LC Column (50 mm × 2.1 mm, 1.7 μm particle size) (Waters, Milford, Massachusetts, United States). The column oven and autosampler cooler were set at a temperature of respectively 45°C and 10°C.

The method uses a gradient elution with an aqueous buffer at pH 9 (solvent A) and methanol (solvent B): 0–10 min: 25–90%, 10–11 min: 90%, 11–11.5 min: 25%, 11.5–13 min: 25%. The system was kept at starting conditions for 5 min to re-equilibrate. The total analytical run time was 13 min. The flow rate was set at 0.5 mL/min with an injection volume of 10 µL.

A triple quadrupole MS (3200 QTRAP, Sciex Halle) was operated in scheduled multiple reaction monitoring mode in combination with a Turbo V ion source with positive electrospray ionization (Sciex, Halle, Belgium). Following source parameters were set: curtain gas: nitrogen, 25 psi; nebulizing gas: nitrogen, 55 psi; heater gas: nitrogen, 55 psi; ion source temperature: 550°C; ion source voltage: + 5,500 V. MRM transitions, retention times and MS parameters are presented in Supplementary Table S2. These MS parameters were determined by direct infusion. The mass spectrometer was coupled to a Dell Precision™ 390 Workstation equipped with Analyst software version 1.5.1. (Sciex, Halle, Belgium) for data acquisition. The screening method is validated and commercially available as iMethod™ Test for Cliquid Software (Sciex, Halle, Belgium).

### Method validation

The method is fully validated for bone tissue and BM as prescribed by international guidelines (23). The following parameters were assessed: selectivity, linearity, matrix effect, recovery, limit of quantification (LOQ), limit of detection (LOD), precision, accuracy and stability. For the validation, blank bone tissue and blank BM were used. The blank bone and BM were taken from forensic cases, which tested, negative for all 415 compounds using a screening method on blood that is described in Vandenbosch et al. (21). Selectivity was tested by analyzing five zero samples from five different donors. LOQs were set as the lowest points of the calibration curve, which fulfilled the criteria of sufficient precision and accuracy using spiked quality control samples. The LODs were estimated using a linear calibration curve containing negative controls *n* = 2, LOQ *n* = 5 and the second lowest calibrator *n* = 5 as described by Polettini et al. (24). Matrix effect was evaluated by testing a methanolic standard A and post-extraction spiked sample B at two concentrations low and high using samples from five different donors as described by Matuszewski et al (25). Recovery was evaluated by testing a methanolic standard (A) and pre-extraction spiked sample (C) at two concentrations (low and high) using samples from five different donors as described by Matuszewski et al. (25). Processed sample stability was tested by analyzing two samples at high and low concentration after 72 h of storage in the 10°C cooled autosampler.

#### Bone tissue and bone marrow

Matrix-matched calibration curves were created using skeletal tissue from five different donors. For all analytes, calibration curves were created (1, 5, 50, 500, 1,000, 2,000, 3,000 and 4,000 ng/g) (*n* = 5 at all concentrations). Different regression models were evaluated: linear least squares un-weighted and weighted (1/*x*, 1/*x*^2^) regression models and quadratic least squares un-weighted and weighted (1/*x*, 1/*x*^2^) regression models. The best calibration models were selected based on the lowest back-calculated values. For all analytes, deuterated standards were available and used as IS with exception of O-desmethyltramadol. For these analytes, cis-tramadol-13C-d_3_ was used as an IS. These ISs were selected based on their similar properties during ionization. Precision and accuracy were evaluated in duplicate on seven different days using quality control samples at low (1 ng/g), medium (500 ng/g) and high concentration (4,000 ng/g). Calibrators were prepared by spiking 100 mg of tissue with different concentrations of methanolic standards. This was done for bone tissue as well as for BM.

#### Blood

Selectivity was tested by analyzing five zero samples from five different donors. For all analytes matrix-matched calibration curves were created (*n* = 5 at all concentration). Different regression models were evaluated: linear least squares un-weighted and weighted (1/*x*, 1/*x*^2^) regression models and quadratic least squares unweighted and weighted (1/*x*, 1/*x*^2^) regression models.

## Results

### Method validation

#### Bone marrow

Blank samples and zero samples showed no interfering peaks for our analytes. Matrix-matched calibration curves were constructed. LODs range from 0.1 to 0.75 ng/g. LOQ has been set as the lowest calibrator, which fulfilled the criteria of sufficient precision and accuracy using spiked quality control samples. Accuracy expressed as bias (%) was in the proposed acceptance limit for all analytes at all concentrations and ranged from −5.86 to 9.65% (23). Repeatability and intermediate precision expressed as relative standard deviations (RSDs) (%) ranged, respectively, from 1.07–8.87% and 0.45–14.93%. All were within the proposed acceptance criteria as prescribed by international guidelines (23). The matrix effects ranged from 66.7 to 105.0%. The recovery ranged from 80.3 to 92.1%. Processed samples were stable in the autosampler, with <10% deviation from starting concentration observed in calculated concentrations up to 72 h post-extraction. Results are summarized in Supplementary Tables S2 and S3.

#### Bone

The method showed no interfering peaks. Matrix-matched calibration curves were constructed. LODs range from 0.1 to 1 ng/g. LOQ has been set as the lowest calibrator, which fulfilled the criteria of sufficient precision and accuracy using spiked quality control samples. Bone concentrations below the LOQ are considered as semi-quantitative. Accuracy expressed as bias (%) was in the proposed acceptance limit for all analytes at all concentrations and ranged from −5.43 to 7.14% (23). Repeatability and intermediate precision expressed as RSD (%) ranged respectively from 0.6–14.4% and 0.5–14.6%. All were within the proposed acceptance criteria as prescribed by international guidelines (23). The matrix effects ranged from 66.5 to 113.0%. The recovery ranged from 76.3 to 96.1%. Processed samples were stable in the autosampler, with <10% deviation from starting concentration observed in calculated concentrations up to 72 h post-extraction. Results are summarized in Supplementary Tables S5 and S6.

#### Blood

No interfering peaks were observed. The best fitted calibration curve showed to be linear for codeine, fentanyl and norfentanyl. For tramadol and O-desmethyltramadol the best fit was linear with weighing factor 1/*x*. The best fitted curve for morphine was with weighing factors of 1/*x*^2^. All curves showed good correlation factors (*R* > 0.99).

### Case studies

#### Routine toxicological screening

Using the iMethod™ Test for Cliquid Software screening method blood, bone and BM of all cases was screened for 415 compounds of forensic interest in positive ion mode. A total of 22 cases were identified with involvement of opioids. In 12 cases tramadol was detected. For codeine, 12 cases were screened positive. Morphine was also detected in 11 of these 12 cases. No heroin (diacetylmorphine) could be detected in these cases, but in five cases 6-acetylmorphine (6-AM) could be detected. In five cases (6, 10, 17, 21, 22), 6-AM was detected in blood. In two cases (10, 21), traces of 6-AM were detected in bone tissue. Only in case 10, 6-AM was detected in BM. All of these 22 cases were selected for further analysis using the quantitative methods.

#### Tramadol

A total number of 12 tramadol positive cases (blood) were analyzed. All results are summarized in Table I. Blood tramadol concentrations ranged between 0.3 and 6,800 ng/mL. The blood concentration of case 15 and 18 was outside the linear range and was thus diluted 1/10 using blank donor blood and the dilution factor. Case 15 and 18 showed blood tramadol concentrations that well exceeded the level to cause coma or even death (26). For the other cases, blood tramadol concentrations can be considered as therapeutic and were well above the LOQ with exception of case 8, 11, 20 and 21. The blood concentration of these latter was below the therapeutic threshold. BM tramadol concentration ranged between 1.3 and 2,200 ng/g. The BM tramadol and O-desmethyltramadol concentration of case 15 were also out of our linear range. Therefore, the sample was diluted 1/10 using blank BM and the concentration was back calculated. Bone tramadol concentration ranged between 0.02 and 6,000 ng/g.

**Table I.** Concentrations Found in Each Biological Matrix

Case #	Drugs detected	Blood concentration	Bone concentration	Bone marrow (ng/g)	Ratio blood/bone	Ratio blood/bone marrow
1	Tramadol	81 ng/mL	18 ng/g	91 ng/g	4.4	0.9
	O-desmethyltramadol	15 ng/mL	1.8 ng/g	31 ng/g	8.4	0.5
2	Tramadol	480 ng/mL	69 ng/g	500 ng/g	7.0	0.9
	O-desmethyltramadol	140 ng/mL	4.7 ng/g	25 ng/g	29.6	5.5
3	Codeine	11 ng/mL	0.3 ng/g	5.2 ng/g	32.3	2.0
	Morphine	80 ng/mL	0.8 ng/g	31 ng/g	96.3	2.5
4	Fentanyl	9.2 ng/mL	3.6 ng/g	12 ng/g	2.6	0.8
	Norfentanyl	6.5 ng/mL	1.1 ng/g	1.5 ng/g	5.7	4.3
5	Fentanyl	1.2 ng/mL	n.d.	1.1 ng/g	n/a	1.1
	Norfentanyl	0.4 ng/mL	n.d.	0.2 ng/g	n/a	1.7
6	Codeine	17 ng/mL	1.3 ng/g	12 ng/g	12.9	1.4
	Morphine	180 ng/mL	3.3 ng/g	71 ng/g	55.7	2.6
7	Codeine	33 ng/mL	2.0 ng/g	78 ng/g	16.6	0.4
	Morphine	41 ng/mL	n.d.	99 ng/g	n/a	0.4
8	Tramadol	0.6 ng/mL	0.0	1.3 ng/g	24.2	0.4
9	Tramadol	2,100 ng/mL	270 ng/g	300 ng/g	7.6	0.7
	O-desmethyltramadol	560 μg/mL	98 ng/g	840 ng/g	5.7	0.7
	Fentanyl	7.9 ng/mL	1.4 ng/g	25 ng/g	5.7	0.3
	Codeine	160 ng/mL	29 ng/g	380 ng/g	5.6	0.4
10	Tramadol	130 ng/mL	3.3 ng/g	140 ng/g	40.7	0.9
	O-desmethyltramadol	8.1 ng/mL	0.2 ng/g	33 ng/g	35.1	0.2
	Codeine	120 μg/mL	5.4 ng/g	230 ng/g	22.3	0.5
	Morphine	1,200 ng/mL	20 ng/g	1,800 ng/g	60.5	0.7
11	Tramadol	0.4 ng/mL	0.2 ng/g	4.9 ng/g	1.9	0.1
	O-desmethyltramadol	0.3 ng/mL	0.0 ng/g	1.3 ng/g	6.5	0.2
	Codeine	1,200 ng/mL	140 ng/g	3,600 ng/g	7.9	32.0
	Morphine	26 ng/mL	3.6 ng/g	74 ng/g	7.1	0.3
12	Tramadol	1,700 ng/mL	320 ng/g	1,900 ng/g	5.4	0.9
	O-desmethyltramadol	270 ng/mL	51 ng/g	28 ng/g	5.3	9.9
13	Codeine	5.0 ng/mL	1.0 ng/g	9.4 ng/g	4.8	0.5
	Morphine	52 ng/mL	8.1 ng/g	50 ng/g	6.4	1.0
14	Norfentanyl	2.8 ng/mL	n.d.	2.1 ng/g	n/a	1.3
15	Tramadol	2,500 ng/mL	6,100 ng/g	22,000 ng/g	4.2	1.1
	O-desmethyltramadol	8,900 ng/mL	670 ng/g	6,000 ng/g	13.4	1.5
16	Morphine	1,800 ng/mL	170 ng/g	1,300 μg/g	10.5	1.4
17	Codeine	31 ng/mL	2.1 ng/g	20 ng/g	15.1	1.5
	Morphine	500 ng/mL	38 ng/g	190 ng/g	13.1	2.5
18	Tramadol	6,800 ng/mL	61 ng/g	19 ng/g	1,133.1	3,604.8
	O-desmethyltramadol	2,600 ng/mL	4.6 ng/g	0.5 ng/g	563.4	4,775.5
19	Codeine	25 ng/mL	5.1 ng/g	3.0 ng/g	4.9	8.5
	Morphine	330 ng/mL	21 ng/g	0.0 ng/g	16.0	n/a
20	Tramadol	0.3 ng/mL	1.7 ng/g	4.1 ng/g	0.2	0.1
	O-desmethyltramadol	n.d.	0.2 ng/g	0.3 ng/g	n/a	n/a
21	Tramadol	0.9 ng/mL	2.6 ng/g	1.3 ng/g	0.3	0.7
	Codeine	33 ng/mL	7.9 ng/g	1,400 ng/g	4.2	0.02
	Morphine	310 ng/mL	22 ng/g	12 ng/g	13.9	25.5
22	Tramadol	110 ng/mL	72 ng/g	4.9 ng/g	1.5	22.2
	O-desmethyltramadol	0.2 ng/mL	0.3 ng/g	n.d.	0.8	n/a
	Codeine	14 ng/mL	4.6 ng/g	14 ng/g	1.5	1.0
	Morphine	150 ng/mL	25 ng/g	96 ng/g	3.0	1.6

n.d. = Not detected.

The metabolite O-desmethyltramadol was present in all tramadol positive cases with exception of 8, 21 and 22. Blood O-desmethyltramadol concentration ranged from 0.24 to 8,900 ng/mL. Bone O-desmethyltramadol concentration ranged from 0.28 to 660 ng/g. BM O-desmethyltramadol concentration ranged from 0.34 to 6,000 ng/g.

#### Codeine

A total number of 11 codeine positive cases (blood) were identified. Results are shown in Table I. Blood concentrations ranged between 5.0 and 160 ng/mL. The blood concentration of cases 3, 7, 9, 17, 19 and 21 were within the therapeutic range (26). Case 11 was above the threshold to be comatose or even lethal. All other cases were considered to have toxic blood concentrations. Blood concentrations were higher than bone concentration in all cases sampled. Bone tissue concentrations ranged between 0.33 and 29 ng/g. Only the bone concentration of case 3 was below the LOQ and will be considered as semi-quantitative. BM concentrations ranged between 2.9 and 1,400 ng/g.

#### Morphine

A total number of 11 morphine positive cases (blood) were identified. Results are shown in Table I. Blood concentrations ranged between 9.2 and 1,700 ng/mL. Multiple cases (6, 10, 16, 19, 21) were above the therapeutic threshold. BM concentrations ranged between 12 and 1,800 ng/g. Bone tissue concentrations ranged between 0.8 and 170 ng/g. The bone concentration in case 3 was below our LOQ. This value will be interpreted semi-quantitative.

#### Fentanyl

A total number of four cases were analyzed where fentanyl was involved. Results are shown in Table I. Blood fentanyl concentration ranged from 1.2 to 9.2 ng/mL. Bone fentanyl concentration ranged between 1.4 and 3.6 ng/mL. BM fentanyl concentration ranged between 1.1 and 25 ng/g.

Blood norfentanyl concentration ranged between 0.4 and 6.9 ng/mL. Bone norfentanyl concentration was two times 1.1 ng/g. BM norfentanyl concentration ranged from 0.21 and 490 ng/g.

## Discussion

Previous studies showed the sample collection procedures of skeletal tissue to be of the most utter importance since sampling location plays a major role in drug concentration (18). However, the sample collection has not been standardized between labs. In a previous study, it was shown that a possible correlation may exist between bone, BM and blood concentration for methadone (21). The current study aims to extend this knowledge to other opioids. Therefore, samples were taken in the same standardized method as described in Vandenbosch et al. (21). Postmortem intervals are reported in Supplementary Table S1. Since PMI are most of the time less than 48 h with a few exceptions and no clear signs of decomposition have been described by the forensic pathologist with exception of one case, the samples in this study can be considered as fresh. Therefore, caution is advised when interpreting skeletal tissue drug concentrations found in heavily decomposed remains. As decomposition proceeds, bones may be soaked in the decomposition fluids from tissues with a high drug concentration (e.g., liver and lung). Due to its more porous structure, spongy bone may be more susceptible to this type of contamination. Furthermore, very little information is known about acute or chronic drugs usage of the deceased. It is possible that uptake into bone is slower than into most tissues. As a result, the bone concentration may not reflect acute overdoses as distinct from long-term therapeutic use leading to a slow accumulation of drug in this tissue. When looking at the case reports, very often polypharmacy is detected. Since no information is available about co-ingestion of certain medication over a period of time or at the same time, caution is advised when interpreting the obtained results. The consequences on the obtained results should be further investigated. This applies to all obtained results in this project. Nevertheless, the findings presented here, pose a starting point toward a better understanding of drug concentration in skeletal tissue and give rise to a starting frame of reference data to conduct toxicological analysis on skeletal tissue when other matrices are unavailable. Some measurements were outside the dynamic range of the calibration curve. The results from these samples should be interpreted as semi-quantitative.

### Tramadol

Apart from case 15 and 18, tramadol drug concentration was always the highest in BM. Blood concentrations exceeded bone concentration with the exception of case 20 and 21. For O-desmethyltramadol, it is more variable which specimen showed a higher concentration. When the drug-to-metabolite relationship is assessed, the ratios were highly variable. Drug-to-metabolite ratios are shown in Supplementary Table S7. But for all cases sampled, the concentration of the more polar metabolite is lower compared to its parent molecule. These ratios are also an indication that measured drug concentrations are not measured from residues of blood. If this was the case a higher concentration of metabolites would be expected similar to those seen in blood. As a result, drugs inside bone tissue may be a distinct compartment.

**Figure 1.**
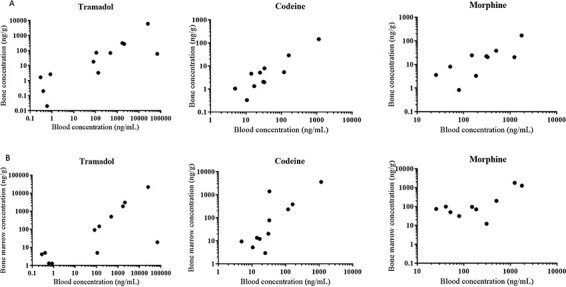
Results based on different postmortem cases. (A) Relation between bone concentrations and blood concentration for each compound. (B) Relation between bone marrow concentrations and blood concentrations for each compound.

This explanation is supported when looking at the trend between blood tramadol concentration and bone concentration. A linear trend can be seen. This is shown in Figure 1A. When looking at the trend between blood and BM concentration, a similar linear trend can be seen. This is shown in Figure 1B. In this trend, case 8 and 18 fall slightly outside the linear trend. Case 18 had blood concentrations well above the toxic threshold. This may explain the outlier. Tramadol concentration in BM may be a delayed representation of blood concentration. A similar thing was described for diazepam in rat studies (27). These results give a good indication of the broad range in which skeletal tissue concentration can be found.

### Codeine

When correlating blood with BM codeine concentration, a linear trend is seen. Results are shown in Figure 1B. Case 21 is identified as an outlier. A higher concentration of codeine was found in BM as expected.

When looking at the trend between blood concentrations and bone concentrations, a linear trend can be seen. Results are shown in Figure 1A. Although a clear trend is visible, variance still exists. An explanation for the discrepancy and high variability could be found in the working mechanism of codeine (28). Codeine is a prodrug. It works on the opioid receptors throughout the body but should first be converted into morphine. Normally around 5–10% of the administered codeine is converted in this way. However, a high inter-individual variability exists.

### Morphine

When the concentrations in this project are compared with a similar case report from Raikos et al (16)., similar BM concentrations are seen. Bone concentrations are lower than seen in the case report by Raikos et al. (16). This could be due to a different extraction solvent and the usage of powdered bone instead of rings. They also suggest a higher morphine concentration due the hydrolysis of 6-AM.

When correlating blood concentration to bone concentration, a linear trend can be seen. This is shown in Figure 1A. When correlating blood concentration to BM concentration, a lot of variability is seen. This is shown in Figure 1B. Still a linear trend could be distinguished. The high variability that is seen, can be explained in a similar trend as for codeine. Since codeine is partly converted into the active morphine, it is not possible to determine which morphine comes from the codeine metabolism. When looking at the results in this project also morphine was detected in every case involving codeine with exception of case 9. The drug-to-metabolite ratio showed to be highly variable. Drug-to-metabolite ratios are shown in Supplementary Table S8. Another possibility is that the detected morphine and codeine are metabolites from heroin usage. This was checked using the screening protocol for heroin and its metabolite 6-AM (29). Case 10 was the only case were 6-AM was detected in BM. Studies have also shown that blood concentrations of codeine are several times higher than morphine after intake of codeine (30). According to forensic recommendations, a morphine-to-codeine value below 1 is considered to be indicative for sole codeine intake, whereas values above 1 are considered to indicate use of heroin (31). When looking at the blood results in our study, the ratios morphine over codeine appear to be high with exception of cases 9 and 11. The ratios are shown in Supplementary Table S8. Cases 9 and 11 show a morphine to codeine ratio that is below 1. Co-ingestion of morphine with heroin and codeine could not be ruled out as a contributing source to measured morphine. This can also account for the variability.

When analyzing the ratios in bone tissue, similar results are obtained as in blood. The morphine-to-codeine ratio was above 1 in all cases with exception of case 9 and 11. So, the morphine-to-codeine ratio in bone tissue may also be valuable to determine heroin usage or codeine ingestion.

When looking at the ratios in BM, the ratios were also above 1 except for cases 11 and 21. In case 21, the ratio is different than in blood or bone tissue. In the BM of this case, the ratio of morphine-to-codeine is well below 1. This may be due the advanced stage of decomposition of the body (32). Since the cases presented here show postmortem intervals ranging from 12 to 168 h, this factor should be considered and further investigated.

### Fentanyl

As far as the author knows, this is the first-time fentanyl is detected and quantified in bone tissue. Fentanyl is a 100 time more potent opioid than morphine. As a result, therapeutic blood concentrations are rather low. Case 5 showed a fentanyl level below the therapeutic threshold. Fentanyl and norfentanyl were undetected in bone tissue of case five. BM fentanyl concentrations were higher than blood in 2 out of 3 cases. This may prove useful to detect fentanyl when blood is unavailable or concentrations are below the LOD. Blood fentanyl concentrations and BM fentanyl concentrations were higher than bone concentrations. The drug-to-metabolite relationship showed to be highly variable for all matrices analyzed. Fentanyl was always present in a higher concentration than norfentanyl with exception of the BM of case 9. In case 14 only norfentanyl could be detected and no fentanyl.

## Conclusion

An LC--MS-MS method was successfully optimized and validated for the analysis of six opioids in skeletal tissue. As far as the author knows, for the first-time codeine, tramadol, O-desmethyltramadol, fentanyl and norfentanyl concentrations were determined in postmortem bone and BM of 22 forensic cases. Furthermore, the morphine concentrations were also determined in bone and BM. When plotting blood concentration of tramadol against bone and BM concentrations, a linear trend could be seen. The same trend was seen when codeine blood concentrations were plotted against their corresponding bone and BM concentrations. However, cases with excessive overdose blood concentrations were clear outliers from these trends. For fentanyl and its metabolite norfentanyl, no conclusions could be drawn because of the small sample size. The results presented here show what concentrations of tramadol, codeine and morphine might be found in the clavicle and its BM. All these findings pose a starting frame of reference to conduct toxicological analysis on skeletal tissue.

## Supplementary Material

bkab095_Supp
