# Supplementary material for: Postmortem Analysis of Opioids and Metabolites in Skeletal Tissue
Source: J Anal Toxicol. 2021 Sep 5;46(7):783–90. doi: 10.1093/jat/bkab095 (PMC9375233; doi:10.1093/jat/bkab095)
Supplement: bkab095_Supp [file bkab095_supp.zip › jat-21-3417-File004.doc]

## Supplementary Tables

| **Case #** | **Age** | **Sex** | **PMI (h)** | **Background** |
| --- | --- | --- | --- | --- |
| 1 | 28 | M | 48 | A 28 year-old man with a history of drug abuse was found dead in his own house. Next to him, some dry white powder was found. In the house, there were also a package of flunitrazepam present. |
|  |  |  |  |  |
| 2 | 48 | M | 36-48 | A 48-year-old man with hyperthermia was found next to a jacuzzi. |
|  |  |  |  |  |
| 3 | 32 | M | 36 | After drinking and using cocaine, the next morning a 32-year-old man was found dead in his couch . In proximity of the couch, burned aluminum foil, ammoniac and a bottle with the label "Methadone" was found. |
|  |  |  |  |  |
| 4 | 58 | M | 24 | After using cocaine and alcohol the day before, a 58-year-old man was found dead in a bathtub. On the body a plaster of Durogesic® was found. |
|  |  |  |  |  |
| 5 | 71 | F | 72 | A 71-year-old woman was found dead in a swimming pool with a fentanyl (25µg/h) plaster on her back. She was in the starting stages of decomposition. |
|  |  |  |  |  |
| 6 | 43 | M | 24-36 | A 43-year-old man was found dead in bed. In his house, cannabis grinders and diazepam were found. In a trashcan, a burned foil of aluminum was found. |
|  |  |  |  |  |
| 7 | 39 | M | 48 | A 39-year-old man with a history ofchronic cocaine usage was found death in a private club. On his clothes, white powder was found. |
|  |  |  |  |  |
| 8 | 21 | M | 48 | A 21-year-old man with a history of substance abuse was found death. In his apartment, multiple drug paraphernalia were found. |

| **Case #** | **Age** | **Sex** | **PMI (h)** | **Background** |
| --- | --- | --- | --- | --- |
| 9 | 45 | F | 24-36 | A 45-year-old woman was found dead in bed. She had history of alcohol and drug abuse. Autopsy showed signs of speeded decomposition. |
| 10 | 25 | M | 24 | A 25-year-old man was found in bed. In the proximity a brown powder was found. |
| 11 | 32 | M | <24 | A 32-year-old man was found dead. He had chronic pain problems with extensive polypharmacy. In his house, tramadol, bromazepam, escitalopram, laxativa, viagra and injection needles were found. |
| 12 | 29 | F | 24-36 | A 29-year-old woman was found hanging from her ceiling. At the scene, alcohol, benzodiazepines and tramadol were found. |
| 13 | 43 | M | 24 | A 43-year-old man was found dead in his room at a psychiatric institution. He had history of drug abuse. |
| 14 | 28 | F | 24 | A 28-year-old woman was found dead in the couch. In the house, methadon, burned alumnium foil, bromazepam, valium and tranxene were found. |
| 15 | 63 | F | 96 | A 63-year-old woman was found dead in a bath. In proximity empty blisters of tramadol and furandatine were found. |
| 16 | 83 | F | 24-48 | An 83-year-old woman got hit by a car and broke her neck |
| 17 | 49 | M | 72-96 | A 49-year-old man was found dead the day after using heroin. He had a history of substance abuse. Next to him, white and brown powder were found. |
| 18 | 23 | M | 48-72 | A 23-year-old man was dead in bed. He was diagnosed with Crohn’s disease and depression. In his room, tramadol, venlafaxine, mirtazapine and zolpidem were found. |
| 19 | / | M | 48-72 | A male was found dead in his couch. In the proximity, multiple drugs paraphernalia were found. He had a history of substance abuse. |
| 20 | 82 | M | 36-48 | An 82-year-old man was found hanging from the ceiling. In his house, lixiana, asaflow, tramadol, zaldiar, bisopropol and metformine were found. |
| 21 | 25 | M | 36-168 | A 25-year-old man was found dead in a squatted building. Autopsy showed the body to be in an early stage of decomposition. |
| 22 | 40 | M | 36 | A 40-year-old man was found dead on his back in a hotel room. He was supposed to meet up with prostitutes and a friend to do drugs. |

***Supplementary Table 1 medico-legal history of each case as provided by the legal system***

|  | Rt | DP | EP | CEP | Q1 mass | Q3 mass | | CE | Q3 mass | CE |
| --- | --- | --- | --- | --- | --- | --- | --- | --- | --- | --- |
|  | (Min) | (V) | (V) | (V) | (Da) | MRM 1 (Da) | | (V) | MRM 2 (Da) | (V) |
| **Tramadol**  **tramadol-c13-d3** | 4.28  3.22 | 26  26 | 4.5  4.5 | 22  20 | 264.3  318.1 | 58.1  58.1 | 31  27 | | 42.0  44.0 | 117  33 |
| **O-Desmethyltramadol** | 3.10 | 36 | 4.0 | 20 | 318.1 | 58.1 | | 27 | 44.0 | 33 |
| **Codeine**  **Codeine-d3**  **Morphine**  **Morphine-d3** | 3.26  3.19  1.85  1.78 | 46  46  81  46 | 5  5.5  5.5  6 | 20  20  18  26 | 325.0  331.0  286.2  314.1 | 262.1  262.1  152.0  262.1 | | 27  27  85  27 | 109.0  109.0  128.0  109.0 | 33  33  33  87 |
| **FENTANYL** | 7.14 | 56 | 7.5 | 14.0 | 337.3 | 105.0 | | 47 | 188.1 | 29 |
| **Fentanyl-d5** | 7.12 | 51 | 5 | 20.0 | 342.3 | 105.0 | | 46 | 188.1 | 30 |
| **NORFENTANYL** | 2.14 | 46 | 10 | 16.0 | 233.2 | 84.1 | | 25 | 115.1 | 90 |
| **NORFENTANYL-d5** | 2.07 | 46 | 10 | 18.0 | 235.2 | 84.1 | | 25 | 115.1 | 90 |

*Underlined transitions were used for quantification. DP: declustering potential; EP: entrance potential; CEP: collision cell entry potential.

***Supplementary Table 2*** Analyte and deuterated internal standard MS-settings and MRM transitions

|  |  | |  |  |  | **Low** | | | | **Medium** | | | | **High** | | | | | |
| --- | --- | --- | --- | --- | --- | --- | --- | --- | --- | --- | --- | --- | --- | --- | --- | --- | --- | --- | --- |
|  | Range (ng/g) | Calibration model | R² | LOD (ng/g) | LOQ (ng/g) | Bias (%) | RSD_rep_ (%) | | RSD_int_ _pr_ (%) | Bias (%) | RSD_rep_ (%) | | RSD_int_ _pr_ (%) | Bias (%) | | RSD_rep_ (%) | RSD_int pr_ (%) |  |  |
| Tramadol | 1-4000 | linear 1/X² | 0.99 | 0.1 | 1 | 9.62 | | 8.87 | 2.01 | 1.25 | 5.28 | 14.88 | | -1.04 | 2.14 | | 14.93 |  | |
|  |  |  |  |  |  |  |  |  |  |  |  |  |  |  |  |  |  |  | |
| O-desmethyltramadol | 1-4000 | linear 1/X² | 0.98 | 0.1 | 1 | 4.42 | | 9.6 | 3.19 | -3.93 | 4.73 | 9.3 | | 1.16 | 2.45 | | 12.49 |  | |
|  |  |  |  |  |  |  |  |  |  |  |  |  |  |  |  |  |  |  | |
| Morphine | 1-4000 | linear 1/X² | 0.99 | 0.5 | 1 | -1.49 | | 3.29 | 1.15 | -5.86 | 3.81 | 7.01 | | -0.82 | 2 | | 12.89 |  | |
|  |  |  |  |  |  |  |  |  |  |  |  |  |  |  |  |  |  |  | |
| Fentanyl | 1-4000 | linear 1/X² | 0.98 | 0.75 | 1 | 2.01 | | 1.07 | 0.47 | -2.69 | 1.22 | 6.12 | | -3.89 | 1.94 | | 12.7 |  | |
|  |  |  |  |  |  |  |  |  |  |  |  |  |  |  |  |  |  |  | |
| Norfentanyl | 1-4000 | linear 1/X | 0.99 | 0.1 | 1 | -0.09 | | 4.49 | 0.94 | -3.47 | 1.29 | 7.06 | | 0.4 | 2.24 | | 13.9 |  | |
|  |  |  |  |  |  |  |  |  |  |  |  |  |  |  |  |  |  |  | |
| Codeine | 1-4000 | linear 1/X² | 0.990 | 0.5 | 1 | 4.84 | | 1.91 | 0.45 | 0.86 | 1.96 | 6.6 | | 6.11 | 2.52 | | 13.79 |  | |
|  |  |  |  |  |  |  |  |  |  |  |  |  |  |  |  |  |  |  | |

***Supplementary Table 3 Validation parameters of the analytical method for bone marrow with coefficients of determination (R^2^), limits of quantification (LOQ), limits of detection (LOD), accuracy estimations as bias and precision presented as relative standard deviation values of repeatability (RSD_rep_) and intermediate precision (RSD_int pr_)***

|  | **Low** | | | | **Medium** | | | | **High** | | | |
| --- | --- | --- | --- | --- | --- | --- | --- | --- | --- | --- | --- | --- |
|  | ME (%) | RSD_ME_ (%) | RE (%) | RSD_RE_ (%) | ME (%) | RSD_ME_ (%) | RE (%) | RSD_RE_ (%) | ME (%) | RSD_ME_ (%) | RE (%) | RSD_RE_ (%) |
| Tramadol | 81.9 | 11.2 | 80.3 | 9.4 | 90 | 13.4 | 90.5 | 6.5 | 105 | 0.9 | 91.1 | 1.1 |
|  |  |  |  |  |  |  |  |  |  |  |  |  |
| O-desmethyltramadol | 82.9 | 14.8 | 81.1 | 7.2 | 99.5 | 3.9 | 89.6 | 8.4 | 105 | 1.8 | 90.5 | 2.5 |
|  |  |  |  |  |  |  |  |  |  |  |  |  |
| Morphine | 84.3 | 12.8 | 85.1 | 8.0 | 67.2 | 7.8 | 86.4 | 7.2 | 69.7 | 7 | 92.1 | 8.3 |
|  |  |  |  |  |  |  |  |  |  |  |  |  |
| Fentanyl | 98.2 | 8.0 | 92.1 | 5.3 | 103.2 | 13.8 | 88.4 | 10.2 | 98 | 7.6 | 88.1 | 7.7 |
|  |  |  |  |  |  |  |  |  |  |  |  |  |
| Norfentanyl | 84.3 | 14.9 | 82.1 | 11.1 | 76.3 | 4.6 | 84.8 | 3.2 | 87.5 | 9 | 84.5 | 7.4 |
|  |  |  |  |  |  |  |  |  |  |  |  |  |
| Codeine | 66.7 | 14.3 | 82.3 | 12.4 | 88.1 | 9.9 | 85.9 | 8.2 | 70.4 | 9.3 | 86.4 | 8.1 |
|  |  |  |  |  |  |  |  |  |  |  |  |  |
|  |  |  |  |  |  |  |  |  |  |  |  |  |
|  |  |  |  |  |  |  |  |  |  |  |  |  |

***Supplementary Table 4 Validation parameters of the analytical method for bone marrow with Matrix effects (ME) and Recovery (RE)***

|  |  | |  |  |  | **Low** | | | **Medium** | | | **High** | | |
| --- | --- | --- | --- | --- | --- | --- | --- | --- | --- | --- | --- | --- | --- | --- |
|  | Range (ng/g) | Calibration model | R^2^ | LOD (ng/g) | LOQ (ng/g) | Bias (%) | RSD_rep_ (%) | RSD_int_ _pr_ (%) | Bias (%) | RSD_rep_ (%) | RSD_int_ _pr_ (%) | Bias (%) | RSD_rep_ (%) | RSD_int_ _pr_ (%) |
| Tramadol | 1-4000 | Linear | 0.996 | 0.1 | 1 | 7.14 | 3.46 | 0.63 | -6.58 | 3 | 8.2 | 1.95 | 1.2 | 15 |
|  |  |  |  |  |  |  |  |  |  |  |  |  |  |  |
| O-desmethyltramadol | 1-4000 | Linear | 0.997 | 0.1 | 1 | -3.76 | 5.41 | 1.1 | -7.71 | 2.5 | 4.4 | 1.35 | 3 | 14 |
|  |  | 1/X² |  |  |  |  |  |  |  |  |  |  |  |  |
| Morphine | 1-4000 | Linear | 0.996 | 0.5 | 1 | 0.71 | 8.03 | 1.59 | 0.19 | 7.76 | 15 | 3.2 | 14.4 | 14.4 |
|  |  | 1/X² |  |  |  |  |  |  |  |  |  |  |  |  |
| Fentanyl | 1-4000 | Linear | 0.991 | 1 | 5 | 1.31 | 7.15 | 1.18 | -1.92 | 4.5 | 10.5 | -1.92 | 2.5 | 12.9 |
|  |  | 1/X² |  |  |  |  |  |  |  |  |  |  |  |  |
| Norfentanyl | 1-4000 | Linear | 0.99 | 0.3 | 1 | -5.43 | 7.84 | 1.99 | 6.73 | 3.28 | 12.1 | -3.37 | 2.7 | 14.6 |
|  |  | 1/X² |  |  |  |  |  |  |  |  |  |  |  |  |
| Codeine | 1-4000 | Linear | 0.992 | 0.5 | 1 | 0.02 | 3.05 | 0.5 | -3.37 | 0.6 | 5.5 | 5.2 | 2.8 | 13.4 |
|  |  | 1/X² |  |  |  |  |  |  |  |  |  |  |  |  |

***Supplementary Table 5 Validation parameters of the analytical method for bone with coefficients of determination (R^2^), limits of quantification (LOQ), limits of detection (LOD), ), Matrix effects (ME),***  ***accuracy estimations as bias and precision presented as relative standard deviation values of repeatability (RSD_rep_) and intermediate precision (RSD_int pr_)***

|  | **Low** | | | | **Medium** | | | | **High** | | | |
| --- | --- | --- | --- | --- | --- | --- | --- | --- | --- | --- | --- | --- |
|  | ME (%) | RSD_ME_ (%) | RE (%) | RSD_RE_ (%) | ME (%) | RSD_ME_ (%) | RE (%) | RSD_RE_ (%) | ME (%) | RSD_ME_ (%) | RE (%) | RSD_RE_ (%) |
| Tramadol | 113 | 12.3 | 91.4 | 8.1 | 89.8 | 9.6 | 92.1 | 8.2 | 74.4 | 10.3 | 82.1 | 5.1 |
|  |  |  |  |  |  |  |  |  |  |  |  |  |
| O-desmethyltramadol | 103.2 | 5.0 | 92.1 | 7.1 | 116.6 | 11.0 | 90.4 | 7.1 | 86.1 | 14.7 | 90.1 | 6.8 |
|  |  |  |  |  |  |  |  |  |  |  |  |  |
| Morphine | 70.7 | 10.9 | 78.1 | 9.4 | 79.6 | 13.1 | 82.6 | 11 | 76 | 12.1 | 75.3 | 7.1 |
|  |  |  |  |  |  |  |  |  |  |  |  |  |
| Fentanyl | 92.5 | 4.3 | 95.4 | 5.1 | 72.9 | 8.9 | 74.4 | 6.6 | 72.4 | 11.9 | 77.8 | 8.1 |
|  |  |  |  |  |  |  |  |  |  |  |  |  |
| Norfentanyl | 98.2 | 5.9 | 96.1 | 7.5 | 110.8 | 6.9 | 89.6 | 5.4 | 71.9 | 8.3 | 79.5 | 6.4 |
|  |  |  |  |  |  |  |  |  |  |  |  |  |
| Codeine | 66.5 | 13.6 | 76.3 | 11.1 | 81 | 5.1 | 85.1 | 7.0 | 74 | 9.4 | 80.3 | 7.5 |
|  |  |  |  |  |  |  |  |  |  |  |  |  |

***Supplementary Table 6 Validation parameters of the analytical method for bone with Matrix effects (ME) and Recovery (RE)***

| **Tramadol/O-desmethyltramadol** | | | |
| --- | --- | --- | --- |
| **Case** | **Blood** | **Bone** | **Bone marrow** |
| **1** | 5.4 | 10.3 | 3.0 |
| **2** | 3.4 | 14.6 | 19.6 |
| **8** | n.a. | n.a. | n.a. |
| **9** | 3.7 | 2.8 | 3.6 |
| **10** | 16.6 | 14.3 | 0.2 |
| **11** | 1.3 | 4.6 | 3.7 |
| **12** | 6.2 | 6.1 | 3.7 |
| **15** | 2.9 | 9.2 | 3.7 |
| **18** | 26.6 | 13.2 | 35.3 |
| **20** | n.a. | n.a. | n.a. |
| **21** | n.a. | n.a. | n.a. |
| **22** | 460.7 | 254.2 | n.a. |

***Supplementary Table 7 metabolite ratio for tramadol/O-desmethyltramadol for skeletal tissue as for blood***

| **Codeine/morphine** | | | |
| --- | --- | --- | --- |
| **Case** | **Blood** | **Bone** | **Bone marrow** |
| **3** | 0.13 | 0.40 | 0.17 |
| **6** | 0.09 | 0.40 | 0.17 |
| **7** | 0.80 | n.a. | 0.79 |
| **10** | 0.10 | 0.26 | 0.13 |
| **11** | 44.9 | 40.4 | 48.7 |
| **13** | 0.10 | 0.13 | 0.19 |
| **17** | 0.06 | 0.05 | 0.10 |
| **19** | 0.08 | 0.25 | n.a. |
| **21** | 0.11 | 0.36 | 115.4 |
| **22** | 0.09 | 0.19 | 0.14 |

***Supplementary Table 8 metabolite ratio for codeine/morphine for skeletal tissue as for blood***

**Supplementary SOP:**

***Sample preparation of skeletal tissue***

1. Approx. 4cm of skeletal tissue is taken during autopsy from the proximal clavicular head.
2. The outside of the bone is cleaned using a scalpel and a sanding machine.
3. A ring of 1 cm is serrated 1 cm from the clavicular head.
4. The ring is placed in the self-developed funnel instrument for bone marrow extraction and centrifuged for 15’ at 3500 rpm.

5. 100 mg bone marrow is taken for extraction using SPE as described in Vandenbosch *et al.* (2020).

6. The ring of bone is further cleaned on the inside from soft tissue using a sanding machine.

7. The ring of bone is extracted by submerging in methanol for 72h.

🡪 If the bone shows signs of decomposition, an extra SPE clean-up step is advised after methanol extraction.
